# Serum fatty acid profile in psoriasis and its comorbidity

**DOI:** 10.1007/s00403-017-1748-x

**Published:** 2017-06-05

**Authors:** Hanna Myśliwiec, Anna Baran, Ewa Harasim-Symbor, Piotr Myśliwiec, Anna Justyna Milewska, Adrian Chabowski, Iwona Flisiak

**Affiliations:** 1 Department of Dermatology and Venereology, Medical University of Bialystok, Żurawia str. 14, 15-540 Białystok, Poland; 2 Department of Physiology, Medical University of Bialystok, Białystok, Poland; 3 1st Department of General and Endocrinological Surgery, Medical University of Bialystok, Białystok, Poland; 4 Department of Statistics and Medical Informatics, Medical University of Bialystok, Białystok, Poland

**Keywords:** Psoriasis, Metabolic syndrome, Fatty acid, MUFA, PUFA, SFA

## Abstract

Psoriasis is a chronic inflammatory skin disease that is accompanied by metabolic disturbances and cardio-metabolic disorders. Fatty acids (FAs) might be a link between psoriasis and its comorbidity. The aim of the study was to evaluate serum concentrations of FAs and to investigate their association with the disease activity, markers of inflammation and possible involvement in psoriatic comorbidity: obesity, type 2 diabetes and hypertension. We measured 14 total serum fatty acids content and composition by gas–liquid chromatography and flame-ionization detector after direct in situ transesterification in 85 patients with exacerbated plaque psoriasis and in 32 healthy controls. FAs were grouped according to their biologic properties to saturated FA (SFA), unsaturated FA (UFA), monounsaturated FA (MUFA), n-3 polyunsaturated FA (n-3 PUFA) and n-6 PUFA. Generally, patients characteristic included: Psoriasis Area and Severity Index (PASI), Body Mass Index, inflammatory and biochemical markers, lipid profile and presence of psoriatic comorbidity. We have observed highly abnormal FAs pattern in psoriatic patients both with and without obesity compared to the control group. We have demonstrated association of PASI with low levels of circulating DHA, n-3 PUFA (*p* = 0.044 and *p* = 0.048, respectively) and high percent of MUFA (*p* = 0.024) in the non-obese psoriatic group. The SFA/UFA ratio increased with the duration of the disease (*p* = 0.03) in all psoriatic patients. These findings indicate abnormal FAs profile in psoriasis which may reflect metabolic disturbances and might play a role in the psoriatic comorbidity.

## Introduction

Psoriasis is a chronic inflammatory disease which affects approximately 1–11% of the world’s population [30]. Previously, psoriasis was considered as exclusive skin disease, but many different studies have demonstrated that it is multisystem disorder [16, 17]. Psoriasis is associated with metabolic syndrome, defined as a coexistence of insuline resistance, obesity, hyperlipidemia and hypertension [15]. It has been established that their progression leads to atherosclerotic vascular disease and type 2 diabetes. Particularly patients with severe psoriasis and psoriatic arthritis have an increased risk of cardiovascular mortality that is independent of traditional cardiovascular risk factors [2, 23]. The release of inflammatory molecules and cytokines in psoriasis may play an important role in this association [9]. Psoriasis predispose to metabolic disturbances, and on the other hand obesity can worsen the skin inflammation in psoriasis. Obesity is associated with low-grade chronic inflammation due to deregulation of immune response. Enlarged adipocytes change their secretion pattern into releasing more pro-inflammatory mediators [32]. Accumulating data suggest that reciprocal interactions between the metabolic disturbances and immune system may be a link between the pathogenesis of psoriasis and obesity. Numerous studies demonstrate that the Body Mass Index (BMI) correlates with the onset and severity of psoriasis [31, 33].

Lipid profile disturbances in the psoriatic patients were reported previously. Serum triglycerides, cholesterol, and low density lipoprotein (LDL) were higher in psoriatic patients as compared to healthy controls [1, 11], while high density lipoprotein (HDL) was significantly decreased [11]. In our previous studies, we reported other metabolic disturbances (sphingolipids, lipokain and fatty acid-binding proteins) in psoriatic patients [4, 5, 24].

Circulating free fatty acids (FFAs) are mainly derived from adipose tissue lipolysis and are major energy source for almost all tissues. Their plasma levels are high during fasting and decline after feeding because of anti-lipolytic action of insulin [26]. FFAs are not only the source of energy and the substrates to form cell membranes, but they also play important role as signaling molecules in inflammatory and metabolic pathways. Fatty acid metabolism, affect the Th17 cell function in obese individuals and it plays a key role in psoriasis pathogenesis [12]. FFAs may act as pro- or anti-inflammatatory mediators depending on their length and saturation [32]. The diet rich in polyunsaturated fatty acids (PUFAs) and Mediterranean diet rich in monounsaturated fatty acids (MUFAs) are connected to lower rate of immune diseases [8, 13, 36]. FAs in psoriatic patients can play important role in the development of psoriatic comorbidity. It is well known that higher saturated fatty acid (SFA) intake is associated with increased cardiovascular risk [10]. PUFAs can protect against insulin resistance, cardiovascular disease and the plasma n-3 PUFA are negatively correlated with metabolic syndrome [20].

Taken together, all these data suggest an important role of FA profile in the pathogenesis of psoriasis and its comorbidity.

To our knowledge, serum fatty acid profile prior to any systemic treatment, was not studied in psoriatic patients in relation to skin disease severity and concomitant diseases. Therefore, the aim of the present study was to evaluate serum FAs concentration in exacerbated plaque psoriasis, to assess its correlation with the clinical disease severity, inflammatory markers and metabolic disturbances. In addition, we investigated the association between FAs and their possible involvement in psoriatic comorbidity: obesity, diabetes mellitus type 2 and hypertension.

## Materials and methods

The study was conducted on 85 consecutive patients (28 females and 57 males) with active plaque psoriasis, at median age 53 (19–79 years) and 32 sex- and age-matched healthy controls. None of the patients or controls were under any dietary restriction. The patients did not use any systemic treatment for one month prior to the study. Body Mass Index (BMI) was evaluated as weight/height^2^ (kg/m^2^). Twenty-seven patients (31.7%) had obesity defined as BMI ≥30 kg/m^2^. The history of hypertension and diabetes as well as results of the laboratory tests were collected from hospital records of the patients. Thirteen patients (15.3%) were diagnosed with type 2 diabetes, 29 (34.1%) suffered from hypertension and 9 (10.6%) patients had history of cardiovascular diseases. Sixteen patients (18.8%) had hypercholesterolemia (>200 mg/dl), and 15 (17.6%) hypertriglyceridaemia (>160 mg/dl). Among 13 patients diagnosed as diabetics, three patients used insulin and other used antidiabetic drugs: eight biguanides (metformin) and two glimepiride.

The severity of psoriasis was estimated using Psoriasis Area and Severity Index (PASI) [30]. Patients with other kind of the disease, like pustular psoriasis, erythrodermic psoriasis or psoriatic arthritis were excluded from the study. All biochemical analysis including C-reactive protein (CRP), serum fasting blood glucose (FBG), total cholesterol, HDL, LDL and triglycerides (TG) were performed in the Central Laboratory of our University Hospital Center. All participants gave their written informed consent before the enrollment. The study was approved by the local bioethical committee.

Peripheral blood samples were taken after overnight fast, before starting the treatment from patients and from the control group. After centrifugation, the serum was stored at −80 °C until analyses were performed.

Total serum fatty acids content and composition was measured according to a method by Glaser et al. [18]. Briefly, 100 µl of serum was incubated in 85 °C for 45 min in 1.5 ml of 3 N methanolic HCl containing 2 g/l BHT (2,6-di-tert-butyl-*p*-cresol, antioxidant). Prior to incubation, 100 μl of internal standard mixture (heptadecanoic acid, cholesteryl-heptadecanoate, triheptadecanoate, diheptadecanoate and diheptadecanoyl-phosphatidylcholine; 0.2/2/1.5/0.2/2 per weight, 10 μg of C17:0 total, in chloroform/methanol 2:1) was added to account for methylation and extraction losses. After cooling to room temperature, fatty acids methyl esters were extracted with 0.5 ml hexane, 30 s of vortexing and centrifugation (5 min@3000*g*). A volume of upper organic phase was transferred glass vials and 1 µl of sample was analyzed by gas–liquid chromatography using a Hewlett-Packard 5890 Series II gas chromatograph, a Agilent J&W CP-Sil 88 capillary column (50 m × 0.25 mm I.D.) and flame-ionization detector. The oven temperature was programmed from 130 to 220 °C at 5 °C/min and held at 220 °C for 32 min. Argon was used as carrier gas. The following fatty acid species were identified and quantified according to respective retention times of synthetic standards: myristic (14:0), palmitic (16:0), palmitoleic (16:1n-7), stearic (18:0), oleic (18:1n-9), linoleic (18:2n-6), α-linolenic (18:3n-3), arachidic (20:0), arachidonic (20:4n-6), eicosapentaenoic (20:5n-3), behenic (22:0), docosahexaenoic (22:6n-3), lignoceric (24:0) and nervonic (24:1n-9) acids. The FAs were additionally grouped according to their biologic properties. The percentage of SFAs (myristic acid, palmitic acid, stearic acid, arachidic acid, behenic acid, lignoceric acid) were measured and calculated. Unsaturated fatty acids (UFAs) were divided into MUFAs (palmitoleic acid, oleic acid, nervonic acid), and PUFAs (n-3 PUFAs: α-linolenic acid, eicosapentaenoic acid (EPA), docosahexaenoic acid (DHA) and n-6 PUFAs: linoleic acid, arachidonic acid.

The data analyses were carried out using Statistica 12.0 software. Descriptive statistics were used to show sociodemographic and biochemistry characteristics of the study groups. We examined the distribution of each continuous variable using Shapiro–Wilk test. We have not found the normal distribution of the analyzed variables. Data were presented as median and quartiles (first and third quartile) and percentage when appropriate. The statistical analysis were performed using Mann–Whitney tests. We calculated the Spearman rank correlation coefficients to measure the relationships between the serum FA and metabolic and clinical variables. Results on the level *p* < 0.05 were regarded as significant.

## Results

A total of 85 patients with exacerbated plaque psoriasis (28 females and 57 males) aged 19–53 (mean 49.7 ± 14.4 years) and 32 age- and sex-matched healthy controls were enrolled in the study. The selected clinical, demographic and laboratory data are summarized in the Table 1. Disease duration ranged from 1 to 58 months (mean 18.5 ± 14.4 months). Mean PASI score was 11.4 ± 8.7. In the study group 50 persons (58.8%) had mild psoriasis (PASI <10), 22 (25.8%) had moderate psoriasis (PASI between 10 and 20) and 13 (15.3%) were diagnosed as severe form of the disease (PASI >20). Patients were evaluated according to present psoriatic comorbidity. The mean BMI value of all study group was 28.5 ± 6.3. Twenty-seven patients (31.8%) had obesity (BMI ≥30) and 58 were non-obese (68.2%).

**Table 1:** Clinical and laboratory characteristics of the psoriatic patients and the control group

	Psoriasis (*n* = 85) Median (Q1, Q3)	Control group (*n* = 32) Median (Q1, Q3)
Age (years)	53.0 (41.0; 59.0)	41.5 (37.0; 53.0)
BMI (kg/m^2^)	27.18 (23.89; 31.60)***	23.8 (22.2; 25.9)
Men:women	57 (67%):28 (33%)	21 (65%):11 (35%)
Psoriasis duration (months)	17.0 (6.0; 29.0)	–
PASI	9.00 (5.5; 14.7)	–
Vitamin D (ng/ml)	15.34 (11.53; 21.92)**	21.3 (16.4; 27.0)
CRP (mg/dl)	2.55 (1.15; 5.85)*	2.61 (1.07; 4.90)
FBG (mg/dl)	88 (77; 98)	84 (72; 97)
Cholesterol (mg/dl)	177 (156; 198)*	180 (152; 192)
TG (mg/dl)	109 (79; 149)	105 (82; 153)

Data shown as median and quartiles (Q_1_ first quartile; Q_3_ third quartile) and percentage

*CRP* c-reactive protein, *FBG* fasting blood glucose, *TG* triglycerides, *BMI* Body Mass Index, *PASI* Psoriasis Area and Severity Index

**Fig. 1:**
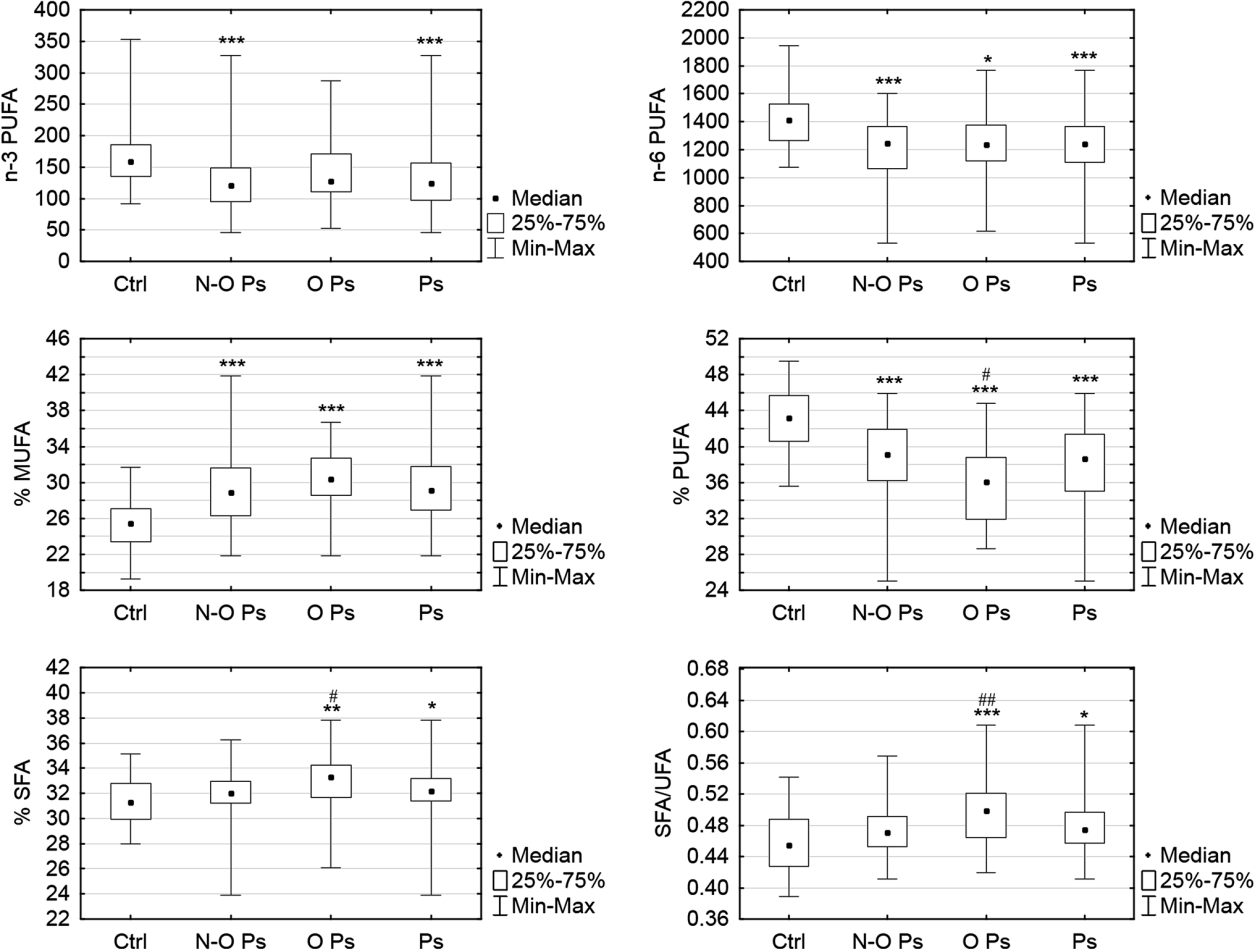
Comparison of FAs pattern in obese psoriatic patients (O Ps), non-obese psoriatic patients N–O Ps), whole psoriatic group (Ps) and the control group (Ctrl). *MUFA* monounsaturated fatty acids, *PUFA* polyunsaturated fatty acids, *SFA* saturated fatty acids, *UFA* unsaturated fatty acids. Data are shown as median and quartiles. Significant differences between the psoriatic groups and the controls are shown as: *p < 0.05; **p < 0.01; ***p < 0.001. Significant differences between the psoriatic subgroups (obese vs non-obese) are shown as ^#^ *p* < 0.05; ^##^ *p* < 0.01

Total FAs concentration is similar in the psoriatic patients and in the control group, but the particular FA is significantly different. FA serum concentration differences between obese, non-obese psoriatic patients and the control group are included in the Table 2. The differences of selected grouped FAs between all psoriatic patient, obese and non-obese subgroups and the control group are shown in the Fig. 1.

**Table 2:** Differences between serum FAs concentrations (mg/l) in obese and non-obese psoriatic patients and the control group

Fatty acid	Psoriasis BMI <30 (*n* = 58)	Psoriasis BMI ≥30 (*n* = 27)	Control (*n* = 32)
Myristic (14:0)	25.3 (19.7–37.4)*	34.8 (22.1–47.2)	34.8 (23.5–56.5)
Palmitic (16:0)	761.2 (706.2–982.2)	882.8 (802.2–1162.4)^#^	755.9 (707.9–959.2)
Palmitoleic (16:1n–7)	97.6 (73.3–154.0)*	124.9 (94.3–155.0)**	75.7 (55.4–101.6)
Stearic (18:0)	233.8 (199.1–268.9)	253.9 (212.2–295.9)	250.5 (233.2–278.4)
Oleic (18:1n9c)	811.9 (717.9–1045.5)	920.5 (807.9–1092.3)*	787.1 (702.8–881.4)
Linoleic (18:2n-6)	964 (840.5–1066.8)***	971.9 (897.2–1117.1)*	1102.5 (1013.5–1229.9)
Arachidic (20:0)	7.3 (6.4–8.8)***	8.5 (7.0–8.9)**	9.9 (8.1–11.5)
α-Linolenic (18:3n-3)	18.5 (16.0–26.5)***	22.4 (19.1–27.4)*	29.8 (23.1–37.1)
Behenic (22:0)	14.6 (13.2–18.0)***	15.8 (13.9–18.7)**	19.5 (16.9–22.5)
Arachidonic (20:4n-6)	249.5 (214.5–288.1)*	266 (230.4–306.6)	285.7 (243.5–315.2)
Lignoceric (24:0)	10.6 (8.8–12.5)***	10.7 (9.5–11.7)***	14.2 (12.1–17.0)
Eicosapentaenoic (20:5n-3)	30.5 (20.9–45.0)**	36.6 (22.8–49.9)	48 (36.4–63.4)
Nervonic (24:1n-9)	43.4 (37.4–49.9)	41.4 (35.1–47.0)	45 (41.6–50.8)
Docosahexaenoic (22:6n-3)	64.7 (53.2–82.3)*	76 (58.9–93.9)	80.8 (67.6–89.9)
Total FA	3417.2 (2991.4–4006.1)	3613.1 (3395.4–4228.0)	3556.1 (3250.8–4147.2)
n-3 PUFA	120.3 (95.3–148.9)***	127.8 (110.6–171.1)	158.7 (135.7–185.7)
n-6 PUFA	1243.3 (1064.6–1367.2)***	1233.3 (1119.2–1375.6)*	1411.3 (1264.9–1529.1)
n-6/n-3 ratio	10.3 (8.4–12.2)	9.3 (7.2–11.3)	9.1 (7.2–10.4)
%SFA	32 (31.2–32.9)	33.3 (31.7–34.3)^**,#^	31.3 (30–32.8)
%MUFA	28.9 (26.3–31.6)***	30.4 (28.6–32.7)***	25.5 (23.4–27.1)
%PUFA	39.1 (36.2–41.9)***	36.1 (31.9–38.8)^***, #^	43.2 (40.6–45.7)
SFA/UFA	0.47 (0.45–0.49)	0.50 (0.46–0.52)^***, ##^	0.45 (0.43–0.49)

Data are shown as median and quartiles (Q_1_ first quartile, Q_3_ third quartile)

Significant differences between psoriatic groups and controls are shown as: * *p* < 0.05; ** *p* < 0.01; *** *p* < 0.001. The differences between the obese and non-obese psoriatic patients as: ^#^ *p* < 0.05; ^##^ *p* < 0.01

**Fig. 2:**
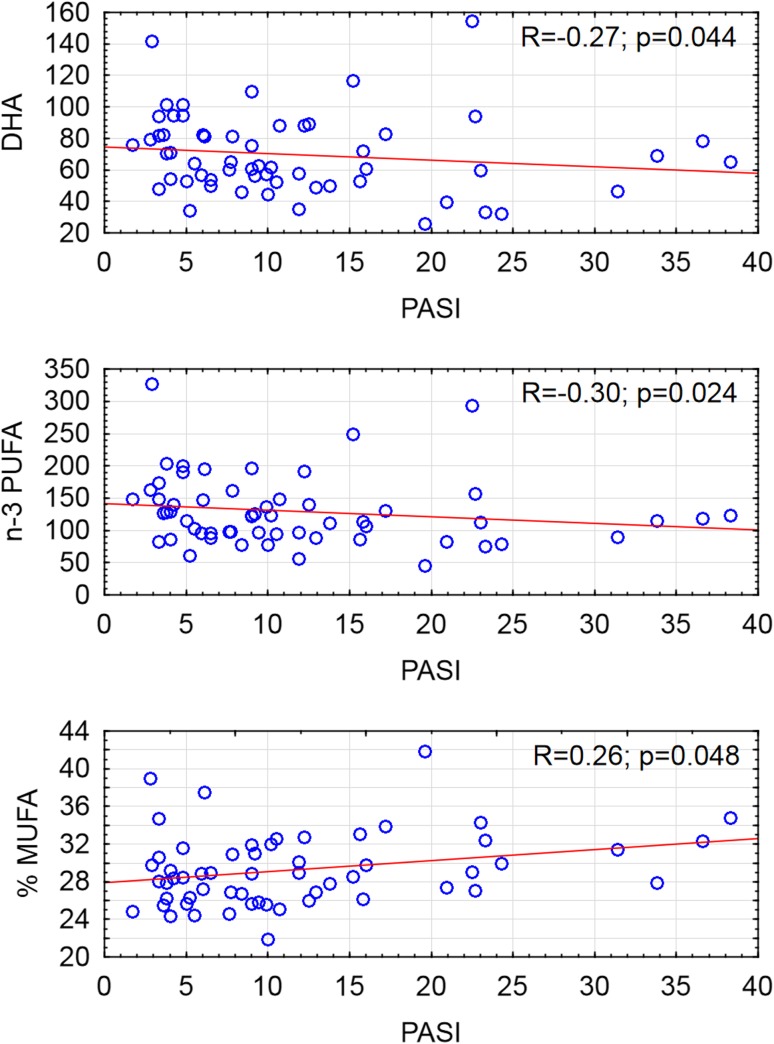
Scatterplot of correlation of docosahexaenoic acid (DHA), n-3 polyunsaturated fatty acid (n-3 PUFA), percent of monounsaturated fatty acid (% MUFA) and PASI in psoriatic patients without obesity (*n* = 58)

We found no correlation between total FA concentration and PASI dependent or independent of BMI. When it comes to particular FA or grouped FA we have shown highly abnormal FA pattern in psoriatic patients. We found a significant negative correlation of EPA and DHA with PASI in the group of all psoriatic patients (respectively: *p* = 0.01; *p* = 0.02). We made similar observations concerning all n-3 PUFAs (*p* = 0.007). Additionally, in the whole group of psoriatic patients, there was a positive correlation between PASI and n-6/n-3 ratio (*p* = 0.012). After the division of our patients into two subgroups: patients with BMI ≥30 (obese) and BMI <30 (non-obese), we found a negative correlation between DHA, n-3 PUFA and PASI, and a positive correlation of the percent of MUFA and PASI in the non-obese psoriatic group (Fig. 2). In the obese psoriatic patients we did not find any significant correlations between FA and PASI. We did not observe any significant correlations between other metabolic parameters such as cholesterol, triglyceride nor fasting blood glucose and PASI in non-obese and obese psoriatic groups.

**Fig. 3:**
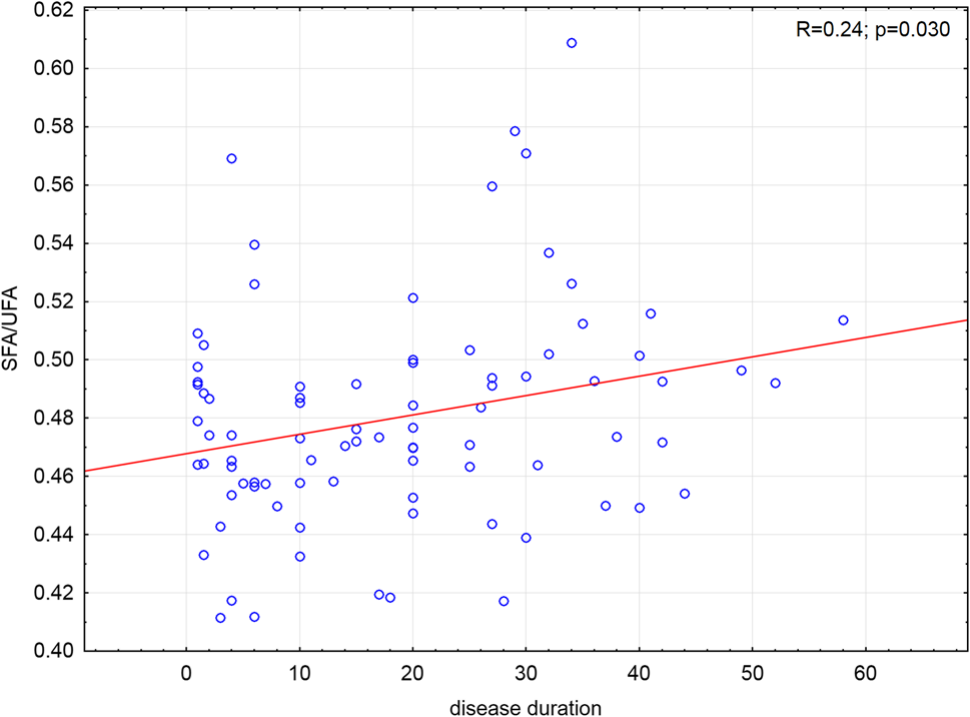
Scatterplot of correlation of saturated to unsaturated fatty acid ratio (SFA/UFA) and duration of the disease (in months) in psoriatic patients (*n* = 85)

Disease duration correlated positively with SFA/UFA ratio (*p* = 0.03) in whole group of patients (*n* = 85) (Fig. 3). After the division in two subgroups: patients with BMI ≥30 and BMI <30 this correlation was not significant.

The results of analysis of correlations (*R* Spearman and *p* value) between particular FA and grouped FA with PASI, disease duration, BMI, CRP, cholesterol, triglyceride, glucose, vitamin D are given separately for non-obese in Table 3 and for obese psoriatic patients in Table 4.

**Table 3:** Correlation of fatty acids with clinical and laboratory data in non-obese psoriatic patients (BMI <30)

Fatty acid	PASI	Disease duration	BMI	CRP	Cholest	TG	FBG	Vit D
Myristic (14:0)	−0.14	0.13	0.30*	−0.18	0.45***	0.59***	0.22	0.14
Palmitic (16:0)	−0.10	0.03	0.28*	−0.16	0.63***	0.62***	0.26	0.07
Palmitoleic (16:1n-7)	0.08	0.05	0.10	0.06	0.31*	0.25	0.05	−0.08
Stearic (18:0)	−0.08	−0.01	0.24	−0.20	0.67***	0.52***	0.39**	0.03
Oleic (18:1n9c)	0.01	0.00	0.21	−0.06	0.53***	0.74***	0.26	0.01
Linoleic (18:2n-6)	−0.13	−0.10	0.17	−0.20	0.75***	0.45***	0.20	0.02
Arachidic (20:0)	−0.10	0.07	0.19	−0.07	0.60***	0.35*	0.45***	0.06
α-Linolenic (18:3n-3)	−0.13	−0.16	0.28*	−0.19	0.37**	0.57***	0.23	0.29*
Behenic (22:0)	−0.12	0.02	0.03	−0.07	0.55***	0.13	0.21	0.02
Arachidonic (20:4n-6)	−0.19	0.12	0.22	−0.25	0.56***	0.32*	0.37**	−0.02
Lignoceric (24:0)	−0.24	0.06	−0.09	−0.29*	0.56***	−0.07	0.10	0.11
Eicozapentaenoic (20:5n-3)	−0.21	0.13	0.31*	−0.27*	0.49***	0.16	0.22	0.37**
Nervonic (24:1n-9)	0.03	0.08	−0.04	0.10	0.50***	−0.02	0.16	−0.03
Docozaheksaenoic (22:6n-3)	−0.27*	−0.07	0.36**	−0.25	0.66***	0.48***	0.36**	0.34**
Total	−0.10	0.01	0.29*	−0.19	0.72***	0.65***	0.29*	0.07
n-3 PUFA	−0.30*	−0.03	0.39**	−0.31*	0.65***	0.45***	0.35*	0.40**
n-6 PUFA	−0.18	−0.05	0.21	−0.26*	0.79***	0.44**	0.26	0.04
n-6/n-3 ratio	0.23	−0.01	−0.33*	0.20	−0.33*	−0.25	−0.21	−0.48***
%SFA	0.01	0.26*	0.08	0.01	0.00	0.09	0.13	−0.08
%MUFA	0.26*	0.04	0.05	0.26	−0.02	0.42**	−0.00	−0.12
%PUFA	−0.20	−0.06	−0.12	−0.28*	0.07	−0.35*	0.00	0.12
SFA/UFA	0.00	0.25	0.08	0.01	0.00	0.09	0.13	−0.08

Spearman’s *R* is shown

The significant correlations are marked as: * *p* < 0.05; ** *p* < 0.01; *** *p* < 0.001

*PASI* Psoriasis Area and Severity Index, *BMI* Body Mass Index, *CRP* C-reactive protein, *Cholest* cholesterol, *TG* triglycerides, *FBG* fasting blood glucose, *Vit D* vitamin D

**Table 4:** Correlation of fatty acids with clinical and laboratory data in obese psoriatic patients (BMI ≥30)

Fatty acid	PASI	Disease duration	BMI	CRP	Cholest	TG	FBG	Vit D
Myristic (14:0)	−0.14	0.11	0.28	−0.04	0.34	0.71***	0.24	0.23
Palmitic (16:0)	0.07	−0.02	0.29	0.08	0.52*	0.81***	0.14	0.31
Palmitoleic (16:1n-7)	−0.11	−0.11	0.21	0.09	0.28	0.61**	0.28	0.01
Stearic (18:0)	0.16	−0.09	0.24	0.00	0.67***	0.56**	−0.01	0.31
Oleic (18:1n9c)	0.16	0.00	0.31	0.09	0.38	0.88***	0.07	0.11
Linoleic (18:2n-6)	0.04	−0.06	0.13	−0.17	0.71***	0.36	−0.28	0.37
Arachidic (20:0)	−0.09	−0.48*	−0.11	−0.04	0.52*	−0.18	0.01	−0.12
α-Linolenic (18:3n-3)	0.03	0.02	0.42*	−0.02	0.57**	0.71***	−0.00	0.11
Behenic (22:0)	−0.23	−0.14	0.01	−0.13	0.55**	−0.22	−0.05	0.10
Arachidonic (20:4n-6)	0.03	−0.40*	0.09	0.04	0.39	0.27	0.15	−0.05
Lignoceric (24:0)	−0.22	−0.14	−0.06	−0.23	0.59**	−0.31	−0.08	0.18
Eicozapentaenoic (20:5n-3)	−0.30	0.00	0.07	0.13	0.51*	−0.06	0.27	0.23
Nervonic (24:1n-9)	0.13	−0.31	−0.03	0.18	0.64**	−0.28	−0.01	0.17
Docozaheksaenoic (22:6n-3)	−0.17	−0.36	−0.01	0.07	0.52*	0.27	0.17	0.18
Total	0.02	0.06	0.32	0.06	0.66***	0.73***	0.09	0.35
n-3 PUFA	−0.21	−0.25	0.08	0.11	0.58**	0.30	0.19	0.18
n-6 PUFA	0.09	−0.14	0.15	−0.12	0.74***	0.42	−0.21	0.29
n-6/n-3 ratio	0.30	0.20	0.00	−0.15	−0.24	−0.06	−0.37	−0.05
%SFA	0.08	0.10	0.09	0.17	−0.23	0.47*	0.29	0.14
%MUFA	0.29	−0.16	0.11	0.26	−0.02	0.75***	0.27	−0.32
%PUFA	−0.05	−0.06	−0.04	−0.09	0.16	−0.62**	−0.34	0.03
SFA/UFA	0.02	0.16	0.08	0.13	−0.25	0.43*	0.31	0.19

Spearman’s *R* is shown

The significant correlations are marked as: * *p* < 0.05; ** *p* < 0.01; *** *p* < 0.001

*PASI* Psoriasis Area and Severity Index, *BMI* Body Mass Index, *CRP* C-reactive protein, *Cholest* cholesterol, *TG* triglycerides, *FBG* fasting blood glucose, *Vit D* vitamin D

Psoriatic patients with hypertension (*n* = 29) compared to psoriatics without hypertension had significantly higher concentrations of total FA (*p* = 0.017), percent of SFA (*p* = 0.031), n-3 PUFA (*p* = 0.007) and SFA/UFA ratio (*p* = 0.014). At the same time they had lower percent of all PUFA (*p* = 0.038) and n-6/n-3 ratio (*p* = 0.005) in serum. Psoriatic group with type 2 diabetes (*n* = 13) compared to non-diabetic patients had significantly higher concentration of total FA (*p* = 0.04) and lower percent of PUFA (*p* = 0.035) Table 5. Psoriatic patients with diabetes were older (*p* = 0.001) and had higher BMI (*p* = 0.1) than psoriatics without diabetes. Similar, psoriatic patient with hypertension were older (*p* = 0.001) and had higher BMI (*p* < 0.001) than those without hypertension (Table 5).

**Table 5:** Differences in FA profile (mg/l) between psoriatic patients with type 2 diabetes, hypertension and patients without comorbidity

	Psoriasis + diabetes	Psoriasis	*p* value
Age	63.0 (56.0–66.0)	52.0 (40.5–57.5)	0.001**
BMI	30.86 (26.5–35.88)	23.61 (30.45)	0.101*
Total FA	3938.5 (3546.2–4331.3)	3453.1 (3078.9–3923.7)	0.040*
%SFA	32.7 (32.0–33.6)	32.1 (31.2–33.0)	0.084
%MUFA	30.7 (29.0–34.1)	29.0 (26.7–31.5)	0.059
%PUFA	34.8 (32.2–38.6)	38.9 (35.3–41.6)	0.035*
SFA/UFA ratio	0.49 (0.47–0.51)	0.47 (0.45–0.49)	0.084
n-3 PUFA	129.0 (113.5–165.9)	122.8 (95.5–149.0)	0.112
n-6 PUFA	1255.0 (1198.1–1335.3)	1237.7 (1085.0–1369.2)	0.526
n-6/n-3 ratio	9.8 (7.0–10.9)	10.2 (8.0–11.9)	0.300

	Psoriasis + hypertension	Psoriasis	*p* value
Age	58.0 (52.0–64.0)	49.5 (37.0–55.0)	0.001**
BMI	31.78 (27.38–36.33)	26.0 (23.4–28.7)	<0.001***
Total FA	3792.4 (3395.4–4228.0)	3395.6 (3018.5–3846.9)	0.017*
%SFA	33.0 (31.4–33.9)	32.1 (31.3–33.0)	0.031*
%MUFA	29.7 (28.4–31.8)	29.0 (26.5–31.7)	0.286
%PUFA	36.6 (33.5–39.2)	39.0 (35.8–41.9)	0.038*
SFA/UFA ratio	0.49 (0.46–0.51)	0.47 (0.45–0.49)	0.014*
n-3 PUFA	146.3 (113.5–171.1)	115.0 (89.4–143.8)	0.007**
n-6 PUFA	1242.0 (1119.2–1386.6)	1238.9 (1051.3–1364.2)	0.528
n-6/n-3 ratio	8.7 (7.2–10.3)	10.5 (8.8–12.4)	0.005**

Data are shown as median and quartiles (Q_1_ first quartile, Q_3_ third quartile)

Significant differences between the groups are shown as: * *p* < 0.05; ** *p* < 0.01, *** *p* < 0.001

*BMI* Body Mass Index, *FA* fatty acid, *SFA* saturated fatty acid, *MUFA* monounsaturated fatty acid, *PUFA* polyunsaturated fatty acid, *UFA* unsaturated fatty acid

## Discussion

We examined circulating FA levels in psoriatic patients with respect to clinical and laboratory data. In the present study the overall concentration of FAs was comparable in psoriatic patients without comorbidity and in the control group but the particular FAs varied greatly (Table 2). We demonstrated a significantly higher percent of MUFA and a lower percent of PUFA in both groups of psoriatic patients (with and without obesity) compared to the healthy control subjects. Our findings may reflect metabolic disturbances of psoriatic patients and explain, at least in part, higher incidence of metabolic syndrome among psoriatic patients than observed in the general population. From the literature we know, that SFA and n-6 PUFA may contribute to proinflammatory state, while MUFA and n-3 PUFA are associated with reduced levels of inflammation [6]. The intake of PUFAs is generally linked also with a reduced cardiovascular disease risk, but an elevated n-6 PUFA intake, without simultaneous n-3 PUFA supply, may increase the risk [22]. Lower of PUFAs observed in our psoriatic group may lead to the proinflammatory state and chronic inflammation.

For the first time, we have found a negative correlation between serum DHA, n-3 PUFAs and the severity of the disease measured by PASI, and a positive correlation between percent of MUFA and PASI (Fig. 2) in non-obese psoriatic patients. We have not observed such correlations in the obese patients. Low concentration of serum n-3 PUFAs in more severe psoriasis may be connected to the inflammatory process. EPA and DHA are the omega-3 fatty acid, which may originate from the diet or can be synthetized from another omega-3 fatty acids e.g. α-linolenic acid. The efficacy of human production of EPA and DHA from ALA is about 5% [7]. EPA and DHA are able to inhibit different mechanism of inflammation, including leukocyte chemotaxis, adhesion molecule expression and leucocyte-endothelial adhesive interactions, production of eicosanoids such as prostaglandins and leukotrienes from the n-6 PUFA arachidonic acid, production of inflammatory cytokines and T cell reactivity [8]. By inhibiting the inflammation they can reduce symptoms of psoriasis [3, 34, 35]. The studies conducted on mice with imiquimod-induced psoriasis-like dermatitis demonstrated that severity of skin lesions was strongly correlated with free fatty acids (FFAs) concentration [32], and obese mice have increased expression of psoriasis mediators, interleukin-17A (IL-17A) and IL-22 in the skin, compared to the control mice [21]. Additionally n-3 PUFAs were shown to have preventing role in psoriasis-like inflammation in mice [27]. In obese psoriatic patients, treated with immunosuppressive drugs, an energy-restricted diet designed to increase n-3 and reduce n-6 PUFAs, ameliorated the metabolic profile and, by increasing the response to immuno-modulating therapy, improved the clinical outcomes of the disease [19]. The important role of n-3 PUFAs in psoriasis pathogenesis was confirmed by a beneficial role of its supplementation. Rahman et al. [28] pointed out even dose-dependent reduction of inflammation and healing of skin lesions after the omega-3 fatty acid systemic treatment. Low concentration of n-3 PUFAs in our psoriatic group may cause more severe skin involvement and systemic inflammation. On the other hand n-3 PUFAs concentration can be the result of some behavioral habits and low consumption of omega-3 fatty acid in general by psoriatic patients which have already been reported [6]. Furthermore, higher n-6/n-3 PUFAs ratio and low serum concentration of EPA and DHA in severe psoriasis, confirmed in our group of patients, may play important role in the development of comorbidity. Several studies in rat and mouse models of induced heart failure have reported that intake of EPA and DHA prevent left ventricular remodeling and contractile dysfunction [14]. Energy-restricted diet enriched in n-3 PUFA has protective effect on metabolic markers and clinical outcome in psoriatic patient with obesity [19]. Several epidemiological studies have confirmed that moderate to severe psoriasis is strongly associated with cardio-metabolic disorders, including hypertension, obesity, type 2 diabetes, dyslipidemia and metabolic syndrome [15]. Our findings stay in line with them and may indicate that in more severe psoriasis there are deeper metabolic disturbances which may increase risk of the metabolic comorbidity.

We have demonstrated a novel association of the duration of the disease with the higher SFA/UFA ratio in all group of psoriatic patients. After the division in two subgroups: patients with BMI ≥30 and BMI <30 this correlation was not significant. Probably the two subgroups are too small to obtain statistical significance of the test. According to these data, the risk of the development of the metabolic disturbances is rising with the psoriasis duration. These findings suggest that chronic inflammation has worse outcome with the prolonged time of duration. Moreover, it is well known from clinical experience that psoriasis which is diagnosed in early age and lasting longer is more likely to have more severe skin lesions and to have higher risk of development of comorbidity in the future. SFA can play a key role in accelerating this process. Additionally, we have observed in our study group higher SFA/UFA ratio in psoriatic patients with obesity and hypertension. The SFA/UFA ratio can be suggested as a marker of cardio-metabolic risk profile of psoriatic patients.

We have discovered that psoriatic patients with obesity and type 2 diabetes have changed FAs profiles due to their metabolic disturbances. Our findings may reflect common chronic inflammatory state of psoriatic patients and insulin resistance. In the presence of adipose tissue insulin resistance, FFA levels are high, despite of high levels of circulating insulin, because of resistance to the anti-lipolytic action of this hormone [26]. Also the patients with hypertension had higher percent of SFA, SFA/UFA ratio, and lower percent of PUFAs. These findings may serve as the missing link in psoriasis comorbidity. Our data are consistent with recent studies of Ni et al. that demonstrate significantly elevated levels of particular FFA in obese patients with type 2 diabetes compared to their healthy counterparts. Moreover, authors have reported that fasting concentrations of similar panel of UFAs were elevated up to 10 years before the onset of metabolic syndrome [25].

The limitation of the study is its retrospective form. We do not know the temporal sequence of the psoriasis and the comorbidity. Inflammatory state of psoriasis could interfere with the adiposity induced inflammation. Psoriatic patients enrolled to the study had no systemic treatment of the psoriasis but continued their medications for other diseases like type 2 diabetes, hypertension and cardiac diseases. This treatment could have interfered with our results. Another limitation is the difference between BMI of psoriatic and control group. Psoriatic patients were more often obese, since the control group consisted of healthy subjects. Abnormal fatty acid profile in the psoriatic patients could be related to psoriasis or coincidental, and we do not know if the FA’s profile is primary or secondary to other immunologic and metabolic disturbances. A prospective study may provide better insight into the association of FA levels with a course of psoriasis and its comorbidity.

In conclusion, in the present study we have shown for the first time the association of circulating FA levels with the metabolic phenotype of psoriatic patients. Our results have additional value in the growing body of evidence indicating a common metabolic profile between psoriasis and metabolic syndrome. We have observed abnormal FAs pattern in psoriatic patients without obesity which may reflect metabolic disturbances in psoriatic patients and higher risk of development of metabolic syndrome. We have demonstrated new association of the severity of the disease with low levels of circulating DHA and n-3 PUFAs. The SFA/UFA ratio was increasing with the duration of the disease. Thus, our findings indicate the abnormal FAs profile in psoriatic patients and in psoriatic comorbidity.
